# Comparison of Oral and Intranasal Midazolam/Ketamine Sedation in 3-6-year-old Uncooperative Dental Patients

**DOI:** 10.15171/joddd.2015.013

**Published:** 2015-06-10

**Authors:** Masoud Fallahinejad Ghajari, Ghassem Ansari, Ali Asghar Soleymani, Shahnaz Shayeghi, Faezeh Fotuhi Ardakani

**Affiliations:** ^1^Associate Professor & Head, Department of Pediatric Dentistry, School of Dentistry, Shahid Beheshti University of Medical Sciences, Tehran, Iran; ^2^Professor, Research Institute of Dental Sciences, Department of Pediatric Dentistry, School of Dentistry, Shahid Beheshti University of Medical Sciences, Tehran, Iran; ^3^Assistant Professor, Department of Pediatric Dentistry, School of Dentistry, Tehran University of Medical Sciences, Tehran, Iran; ^4^Associate Professor, Department of Anesthesiology, Shahid Beheshti University of Medical Sciences, Tehran, Iran; ^5^Assistant Professor, Department of Pediatric Dentistry, School of Dentistry, Shahid Sadoughi University of Medical Sciences, Yazd, Iran

**Keywords:** Anxiety, intranasal, ketamine, midazolam, oral sedation

## Abstract

***Background and aims.*** There are several known sedative drugs, with midazolam and ketamine being the most commonly used drugs in children. The aim of this study was to compare the effect of intranasal and oral midazolam plus ketamine in children with high levels of dental anxiety.

***Materials and methods.***A crossover double-blind clinical trial was conducted on 23 uncooperative children aged 3-6 (negative or definitely negative by Frankel scale), who required at least two similar dental treatment visits. Cases were randomly given ketamine (10 mg/kg) and midazolam (0.5 mg/kg) through oral or intranasal routes in each visit. The sedative efficacy of the agents was assessed by an overall success rate judged by two independent pediatric dentists based on Houpt’s scale for sedation. Data analysis was carried out using Wilcoxon test and paired t-test.

***Results.*** Intranasal administration was more effective in reduction of crying and movement during dental procedures compared to oral sedation (P<0.05). Overall behavior control was scored higher in nasal compared to oral routes at the time of LA injection and after 15 minutes (P<0.05). The difference was found to be statistically significant at the start and during treatment. However, the difference was no longer significant after 30 minutes, with the vital signs remaining within physiological limits. Recovery time was longer in the intranasal group (P<0.001) with a more sleepy face (P=0.004).

***Conclusion.***. Intranasal midazolam/ketamine combination was more satisfactory and effective than the oral route when sedating uncooperative children.

## Introduction

One of the most common challenges faced by pediatric dentists in daily practice is child behavior management. Any impression from a dental experience will be reflected through individual’s future dental attendance by creation of positive or negative memories.^1^ Various approaches have been identified to enable the operator to overcome behavioral problems in children. However, in those with absolute negative behavior, help is necessary to achieve a successful procedure. Even an experienced pediatric dentist may find it difficult to render treatments to certain children when using conventional techniques. In these circumstances the use of conscious sedation and general anesthesia (GA) is considered as helpful alternatives.^2^

It is also important to note that because of the changes in society and the population’s attitude toward interaction with children, older methods of physical restraints such as HOME (hand over mouth exercise) or the use of other physical restraints have lower credibility. In fact, such applications are only limited to certain cases with GA/sedation medical contraindications. Alternative methods of conscious sedation and general anesthesia are ways to overcome the behavioral problems. Several methods have been introduced in this regard, including topical, sublingual, intranasal, rectal, intracutaneous, subcutaneous, intramuscular, intravenous and inhalation routes.^3^ It is important to note that each route has its advantages and disadvantages and could not be considered for every case at every clinical situation.^4^ An example of limitations is the problem with initiation of sedation effect in oral sedation. The oral sedation onset time is long (delayed), while the drug’s absorption level is somehow unreliable. Another major issue in oral sedation is lack of titration capacity and its long-lasting effect delaying patient's discharge.^3^ On the other hand, intranasal sedation is a more recent approach which is considered as one of the alternate ways for prescribing certain medications to the existing oral technique. Intranasal sedation is known as a non-invasive way of drug administration, which is safe and is tolerated by children, with direct absorption potential of the sedative agent into the bloodstream without entering the liver and stomach. It also saves the fearful child from receiving more injections. The level of drug’s absorption is almost similar to that of the IV sedation with peak plasma levels being reached in approximately 10 minutes.^5-6^

For long, conscious sedation has been considered as one of the most reliable alternatives to overcome high levels of interfering dental anxiety with acceptable levels of health and safety of the patient when used by skilled pedodontists. Midazolam and ketamine are two well known sedative drugs with unique characteristics.^7^ Bahetwar et al^8^ showed that ketamine and midazolam are safe and efficient separately and in combination (success rates of 89%, 69% and 84%, respectively). Oral sedation has been satisfactorily achieved by the administration of chloral hydrate and hydroxizine in children.^9^ Submucosal meperidine has also been successfully tested in pediatric dental sedation.^10^

Lee-Kim^11^ compared the intranasal (IN) sedation of midazolam (0.3 mg/kg) and peroral (PO) midazolam (0.7 mg/kg) in pediatric dentistry. The children’s general behavior were shown to be similar in both IN and PO sedation while more child movement and less drowsiness were reported in IV sedation.

The aim of this study was to compare the efficacy and safety of intranasal midazolam/ketamine combination with oral methods along with N_2_O in sedating children for dental procedures.

## Materials and Methods

This randomized crossover double-blind clinical trial (IRCT ID: IRCT201305101882N3) was conducted on 23 children aged 3-6, who were referred to the Department of Pediatric Dentistry, Shahid Beheshti Dental School, Tehran, Iran. Uncooperative 3-6-year-old children with negative and definitely negative Frankel scale in ASA I or II were included in this dental treatment process under conscious sedation.^3^ Cases with at least two similar treatment needs were booked for two separate sessions needing pulpotomy with SSC or restoration. Exclusion criteria were any systemic disease, allergy to drugs, colds, nasal obstruction, respiratory infections, limited neck movement, macroglossia, tonsillar hypertrophy and microglossia. A written informed consent was sought from each patient’s parents with full pre-sedation instructions. All the steps of sedation were performed under the direct supervision of an anesthesiologist. Major vital signs of SPO_2_ respiratory rate and BP were recorded at the start, middle and end of the treatment session. All the children were requested to be kept at NPO status for 6 hours (solid foods) and 4 hours (water and liquids) preoperatively. The children were randomly assigned to two groups for the starting technique of A: intranasal sedation and B: oral sedation. A questionnaire was used to record medical and dental histories. For sedation preparation in group A, 1 mL of 2% lidocaine hydrochloride (Pasteur Industrial Co, Iran) was added to 0.25 mg/kg of atropine (Abu Reyhan Pharmaceutics Co., Tehran, Iran). This combination was then dropped into the nostril mucosa using a 2-mL syringe. A second combination was an 0.5-mg/kg midazolam vial (Chemidaru Industrial Co., Tehran, Iran) added to 10 mg/kg of ketamine (Chemidaru Industrial Co., Tehran, Iran) before being dropped into the nostrils, 5 minutes after the administration of the initial drugs.

The second group received 0.5 mg/kg of oral midazolam (Amsed Syrup, Dales Pharmaceuticals, Skipton, North Yorkshire, UK) along with 10 mg/kg of ketamine and 0.25 mg/kg of atropine in a cup or syringe. Both groups received the alternate treatment protocol during their second session. This was aimed to have every child serve as his or her own control. A local anesthetic was obtained using a 2% lidocaine hydrochloride cartridge with 1:80000 epinephrine (Darupakhsh Pharmaceutical MFG, Co., Iran). Dental treatments were carried out by a pedodontist blinded to the type of the medications administered. The sedative effect of the drugs was measured after 40 and 15 minutes of oral and nasal administrations, respectively. Dissociative state induced by ketamine sedation was judged by semi-closed eyes and nystagmus. A standard flow of N_2_O (40%) and O_2_ was administered for all the patients throughout the treatment courses.

Oxygen saturation was monitored at various steps of the study starting with premedication time at baseline using a multipurpose monitoring unit (Saadat Co, Iran).

All the measurements were made at baseline, at the local anesthetic injection, and 15 and 30 minutes after the start of dental treatment. Houpt Scale was used to record every change in child’s behavior with the following criteria: the amount of crying (C), sleeping (S) and movement (M) and overall behavior (O).^6,12^Video recordings were also scheduled for the entire treatment sessions, which were subjected to evaluation and scoring by an independent pediatric dentist blinded to the administration of drugs. In case of poor cooperation, further sedative drugs were administered if needed in order to complete the treatment process while the least score was recorded for the case and sedation technique. Attempts were made to limit each treatment session to a maximum of 35 minutes.

The children were discharged when full consciousness was achieved as judged by the anesthesiologist and all vital signs returned to normal ranges. The parents were interviewed 24 hours after each session to respond to a series of questions in the patient’s form in relation to postoperative complications. Data were analyzed using Wilcoxon test and paired t-test.

## Results

Data from all the 23 children (18 boys, 5 girls), aged 3-6, were recorded. An initial behavior rating scale (Frankel scale) evaluation revealed that 21 cases (91.3%) were completely negative while 2 (8.7%) cases were judged to be negative. Overall no significant differences were found between the oral and nasal sedation with the drugs administered.

Comparison of sleep (S), movement (M), crying (C) and overall behavior (O) parameters showed significant differences between oral and nasal groups at LA injection time and after 15 minutes (P<0.05) in favor of intranasal sedation. However, these differences were not found to be significant after 30 minutes.

As detailed in the “Materials and Methods” section, all the participants were selected from those classified as definitely negative with the drug administration being carried out by force in both sessions. Since each patient served as control, comparison of the outcomes showed little or no difference in drug acceptance rates. The success rates of oral and nasal administrations at different measured steps showed that the difference was statistically significant after 15 and 30 minutes (P<0.05). These measures were 96.6% and 60.9% for nasal sedation after 15 and 30 minutes versus 39.1% and 34.7% for oral sedation at two occasions, respectively.

According to parents, the most common complications after the treatment were nausea, vomiting, drowsiness and reduction of activity during the initial 24 hours of both oral and nasal sedation sessions.

Recovery time was shorter in oral sedation (1 hour) compared to nasal approach (2.5 hours), indicating a significant difference (P<0.05) between the two.

There was no significant difference between nasal and oral sedation sessions when dental operation was judged. Likewise 87% of parents had a similar impression with little or no difference between the two sedation techniques and sessions. Maximum drowsiness was reported as lasting just under 2 hours following oral intake while this was 4-6 hours in the nasal group with the difference as being statistically significant (P=0.004).

There were no statistically significant differences between the two sedation groups in their heart rates (HR), SPO_2_, RR, and maximum and minimum BP changes.

## Discussion

Based on the results of this investigation there is a promising potential for the use of sedation techniques to overcome children’s interfering behaviors in the dental office. Both intranasal and oral sedations could provide certain levels of calmness for the child and dentist while a dental procedure is underway. Among the two techniques of sedation, it appears that the intranasal method provided a higher and more satisfactory sedation rate, however this difference was not statistically significant. These results enhanced the idea that although behavior management techniques are considered useful tools in controlling most of the uncooperative children, the remaining cases who do not respond to these techniques could benefit from pharmacological aids. These may include various conscious sedation techniques to even full general anesthesia for single-visit full-mouth dental treatment.^8^

Oral sedation is the most common yet easily accepted technique among the various routes of sedation in children. However, delayed onset is considered as the main disadvantage of oral sedation in addition to a long recovery period and high first pass metabolism.^5,9^ The highest level of effect is usually reached after 40-60 minutes of drug administration.^5^

Intranasal administration involves a path in which the drug is administered, aiming to have an immediate absorption into the bloodstream, because of high vascularity of nasal mucosa and increased drug bioavailability without first pass metabolism effect. The technique is simple and effective and requires minimal cooperation.^4,5,13^

In these lines midazolam is considered as the most popular medication which like other benzodiazepines exhibits several positive effects, including hypnotic sedation, amnesia, muscle relaxation and relief of anxiety. Its ability to create anterograde amnesia is much higher when compared to other benzodiazepines.^14^ Higher bioavailability and quicker onset have been demonstrated for intranasal midazolam administration.^8,15^

Ketamine is also another widely used sedative drug with unique properties, including cataleptic condition, amnesia and respiratory and cardiac stability. Its lower doses are routinely used as a sedative in many medical and dental diagnostic and therapeutic procedures.^16,17^

Additional administration of N_2_O-O_2_ alongside benzodiazepines in the form of a cocktail could boost the sedative outcome during child’s dental visit while maintains adequate oxygen levels.^18-20^ Optimal dose for oral ketamine is indicated as 3-10 mg/kg with 0.2-0.75 mg/kg of midazolam in children.^5^ Miller^21^ recommended 6-10 mg/kg of oral and 5-6 mg/kg of nasal ketamine as the safe and effective dose.

Gharde et al^22^ successfully used 10 mg/kg of ketamine and 0.2 mg/kg of midazolam in combination with 0.1 mg/kg of midazolam and 7.5 mg/kg of ketamine intranasally for premedication of children with Tetralogy of Fallot. Tszeet al^23^ evaluated the effect of nasal doses of 3, 6 and 9 mg/kg of ketamine for pediatric sedation in wound healing and reported no sufficient sedation being achieved when INK doses below 6 mg/kgwere used. It was concluded that 9 mg/kg of nasal ketamine is capable of providing 95% sedation rate needed for most of the dental processes. Combination of midazolam and ketamine as oral or nasal medication have been reported to boost the results. Vahid et al^24^ compared oral midazolam (0/4 mg/kg)-ketamine (5 mg/kg) and midazolam (0/5 mg/kg)-promethazine (5 mg/mL) as sedative agents and concluded that ketamine/midazolam combination could sufficiently sedate children even in lower doses, unlike midazolam/promethazine combination used in their study.

Bahetwar et al^8^ compared nasal ketamine and midazolam and their combination with success rates of 98%, 69% and 89%, respectively. It is evident that such nasal combinations could induce moderate sedation, with midazolam remaining to be the weakest when administered alone. In contrast, Lee-Kim^11^ showed no significant differences between the success rates of oral and nasal midazolam. Fuks^25^ compared intranasal 0.2 and 0.3 mg/kg midazolam and showed no significant differences between the outcomes achieved from the doses tested with a generally successful sedation effect achieved, similar to the findings of the current study.

Diaz^26^ reported no nausea and vomiting following the administration of ketamine and placebo, with no post-sedation complications being reported in this investigation with the use of similar sedative agents. No significant differences were noted in the level of cases with nausea, vomiting, headache of the two groups tested. Damle et al^15^ reported higher levels of nausea following the use of ketamine than when midazolam was used.

Ketamine is an analgesic while midazolam is an anti-anxiety and sedative agent but lacks any analgesic effects. In addition to the use of any sedative drugs, the use of local anesthesia is essential for most of the dental treatments.^8,11,24,27^ Postoperative sleep phase was 0-1 hour for oral sedation with 2-3 hours for intranasal sedations at the recovery stage, with statistically significant differences.

## Conclusion

1. The combination of intranasal ketamine-midazolam produced a more satisfactory level of sedation in children for short dental procedures (35 minutes) compared to that of oral route based on Houpt Scales‏.
2. The differences in the overall sedation levels achieved were statistically significant at 15- and 30-minute intervals.

## Acknowledgments

The authors would like to thank the generous scientific and financial support and help of Dental Research Center, Research Institute of Dental Sciences, Shahid Beheshti University of Medical Sciences, Tehran, Iran.
